# Brain volume and neurodevelopment at 13 years following sepsis in very preterm infants

**DOI:** 10.1038/s41390-024-03407-w

**Published:** 2024-07-13

**Authors:** Deanne K. Thompson, Shirley Cai, Claire E. Kelly, Bonnie Alexander, Lillian G. Matthews, Rheanna Mainzer, Lex W. Doyle, Jeanie L. Y. Cheong, Terrie E. Inder, Joseph Y. M. Yang, Peter J. Anderson

**Affiliations:** 1 Victorian Infant Brain Studies, Murdoch Children’s Research Institute, Parkville, VIC 3052 Australia; 2 Developmental Imaging, Murdoch Children’s Research Institute, Parkville, VIC 3052 Australia; 3 Department of Paediatrics, The University of Melbourne, Parkville, VIC 3052 Australia; 4 Turner Institute for Brain and Mental Health, School of Psychological Sciences, Faculty of Medicine Nursing and Health Sciences, Monash University, Clayton, VIC 3800 Australia; 5 Melbourne Medicine School, Faculty of Medicine, Dentistry and Health Sciences, The University of Melbourne, Parkville, VIC 3052 Australia; 6 Neuroscience Advanced Clinical Imaging Service (NACIS), Department of Neurosurgery, Royal Children’s Hospital, Parkville, VIC 3052 Australia; 7 Department of Pediatric Newborn Medicine, Brigham and Women’s Hospital, Harvard Medical School, Boston, MA 02115 USA; 8 Clinical Epidemiology and Biostatistics Unit, Murdoch Children’s Research Institute, Parkville, VIC 3052 Australia; 9 Neonatal Services, The Royal Women’s Hospital, Parkville, VIC 3052 Australia; 10 Department of Obstetrics, Gynaecology and Newborn Health, The University of Melbourne, Parkville, VIC 3052 Australia; 11 Center for Neonatal Research, Children’s Hospital of Orange County, Orange, CA 92866 USA; 12 Department of Pediatrics, University of California, Irvine, CA 92697 USA; 13 Neuroscience Research, Murdoch Children’s Research Institute, Parkville, VIC 3052 Australia

## Abstract

**Background:**

Associations of neonatal infection with brain growth and later neurodevelopmental outcomes in very preterm (VP) infants are unclear. This study aimed to assess associations of neonatal sepsis in VP infants with (1) brain growth from term-equivalent age to 13 years; and (2) 13-year brain volume and neurodevelopmental outcomes.

**Methods:**

224 infants born VP ( < 30 weeks’ gestation/<1250 g birthweight) were recruited. Longitudinal brain volumes for 68 cortical and 14 subcortical regions were derived from MRI at term-equivalent, 7 and/or 13 years of age for 216 children (79 with neonatal sepsis and 137 without). 177 children (79%) had neurodevelopmental assessments at age 13. Of these, 63 with neonatal sepsis were compared with 114 without. Brain volumetric growth trajectories across time points were compared between sepsis and no-sepsis groups using mixed effects models. Linear regressions compared brain volume and neurodevelopmental outcome measures at 13 years between sepsis and no sepsis groups.

**Results:**

Growth trajectories were similar and there was little evidence for differences in brain volumes or neurodevelopmental domains at age 13 years between those with or without sepsis.

**Conclusions:**

Neonatal sepsis in children born VP does not appear to disrupt subsequent brain development, or to have functional consequences in early adolescence.

**Impact statement:**

- Neonatal sepsis has been associated with poorer short-term neurodevelopmental outcomes and reduced brain volumes in very preterm infants.
- This manuscript provides new insights into the long-term brain development and neurodevelopmental outcomes of very preterm-born children who did or did not have neonatal sepsis.
- We found that regional brain volumes up to 13 years, and neurodevelopmental outcomes at age 13, were similar between those with and without neonatal sepsis.
- The links between neonatal sepsis and long-term neurodevelopment remain unclear.

## Introduction

Children born VP are at greater risk for neurodevelopmental impairments than their term-born peers,^1^ but there is wide variability in long-term outcomes.^2,3^ This may be explained by exposure to specific perinatal complications such as white matter injury and chronic lung disease (requiring postnatal corticosteroids), but also more scarcely investigated complications such as neonatal sepsis or necrotising enterocolitis (NEC).^4,5^ We have previously reported poorer psychomotor development at 2 years of age in children born VP with neonatal sepsis, which was mediated by white matter abnormality.^6^ However, the consequences of neonatal sepsis on longer term neurodevelopmental outcomes remain unclear,^7^ with impairments in higher order attention, working memory and executive functions in children born VP becoming more apparent with increasing age.^8^

Magnetic resonance imaging (MRI) can characterise brain dysmaturation that may underpin the perturbed neurodevelopmental outcomes in children born VP. In the newborn period infants born VP have smaller brain volumes compared with infants born at term, and regional brain vulnerabilities persist into childhood.^9–12^ Early perinatal insults, such as sepsis, are associated with reduced cerebral volumes, total brain volumes and intracranial volumes in VP infants.^13,14^ However, the long-term effects of sepsis on the growth of regional brain volumes from term-equivalent age to early adolescence have not been defined.

This study evaluated the brain volumetric changes and long-term neurodevelopmental effects in children born VP with or without neonatal sepsis, using the Victorian Infant Brain Studies (VIBeS) longitudinal cohort, following VP infants from the newborn period, to 7 and 13 years of age. The specific aims of this study were to: (1) describe the mean trajectories of regional brain volume from term equivalent age to 13 years, and (2) assess the impact of neonatal sepsis on brain volume and neurodevelopmental outcomes at 13 years of age. We predicted that neonatal sepsis would be associated with slower brain growth trajectories compared with those without sepsis. We also hypothesised that neonatal sepsis would be associated with smaller regional brain volumes and poorer neurodevelopmental outcomes at 13-years.

## Methods

### Participants

The VIBeS prospective longitudinal cohort study recruited 224 surviving infants who were born <30 weeks’ gestation and/or <1250 g birthweight and admitted to the Royal Women’s Hospital in Melbourne, Australia between July 2001 and 2004. Infants with genetic or congenital abnormalities were not eligible. The study was approved by the Human Research and Ethics Committees of the Royal Women’s and Royal Children’s Hospitals Melbourne. Parents gave written informed consent for their child to participate.

### Sepsis

Infant sepsis was classified in the neonatal intensive care unit as previously described for this cohort.^6^ In brief, infants with confirmed sepsis had: (a) a positive blood culture, and (b) an abnormal total neutrophil ratio ( ≥ 0.12) or abnormal C-reactive protein ( > 8 mg/dL) or abnormal platelet count ( < 100 х 10^9^ /L) within 48 h of the positive blood culture, and (c) at least 5 days of treatment with appropriate antibiotics. Confirmed NEC cases were included in the sepsis group, defined as the presence of at least stage 2a (presence of pneumatosis intestinalis on abdominal x-ray films) of the modified Bell’s classification of NEC.^15^ No infants in our sample had culture-proven meningitis.

### MRI data acquisition

Two-hundred and twenty-four VP infants had MRI scans for the infant time-point. VP children were followed up with scans at 7 years of age (*n* = 159), and 13 years of age (*n* = 141) at the Royal Children’s Hospital Melbourne, as previously described.^16^ A 1.5 Tesla General Electric MRI scanner was used at the infant time-point, and a 3.0 Tesla Siemens Trio MRI scanner at the 7 and 13 year time-points.

### MRI data processing and brain volumetry

Two-hundred and seven of the 224 infants were scanned within the desired window of 38–42 weeks’ gestation (term-equivalent age); the 17 children scanned outside of this window were excluded. Infants’ *T*_*2*_-weighted MRI scans were parcellated using the Melbourne Children’s Regional Infant Brain (M-CRIB) atlas,^17^ as previously described.^12^

At 7 and 13 years of age, regional brain volumes were obtained as previously described,^12^ using FreeSurfer 6.0^18^ parcellation on *T*_*1*_ images, incorporating brainstem and whole hippocampal volumes using FreeSurfer’s subfields tools.^19,20^ The intracranial volume, total brain tissue volume and total cerebrospinal fluid volume were obtained using Statistical Parametric Mapping version 12.^21^

There were 68 cortical and 14 subcortical brain regional volumes of interest across the 3 time-points that were analyzed for this study (listed in Supplementary Tables 1 and 2). All brain volumetry was carried out blinded to sepsis status and was overseen by DKT at all time-points.

### Neurodevelopmental outcomes at thirteen years of age

At 13 years’ corrected age, general intellectual ability (IQ) was estimated with the Composite IQ standard score from the Kaufman Brief Intelligence Test, Second Edition^22^ (KBIT-2). Sustained attention was assessed using the “Score!” standard score from the Test of Everyday Attention for Children^23^ (TEA-Ch). Working memory was assessed using the Backwards Digit Recall standard score from the Working Memory Test Battery for Children^24^ (WMTB-C). Memory and learning were assessed using the California Verbal Learning Test, Children’s Version^25^ (CVLT-C). Overall language functioning was evaluated using the Core Language Score composite index from the Clinical Evaluations of Language Fundamentals, Fourth Edition^26^ (CELF-4). Executive function, in this case planning ability, was assessed with the Tower Test Achievement scaled score from the Delis-Kaplan Executive Function Systems^27^ (D-KEFS). Spelling, reading and mathematics were assessed with standard scores from the Wide Range Achievement Test – 4th edition^26^ (WRAT-4). Behaviour was assessed using the parent reported Total Difficulties Score from the Strengths & Difficulties Questionnaire^28^ (SDQ), where higher scores represent greater behavioural difficulties. Motor skills were assessed using the total test standard score from the Movement Assessment Battery for Children – Second Edition^29^ (MABC-2). Tests used standard scores or composite index scores with a mean of 100 and a standard deviation (SD) of 15, except for tests of memory and executive function which generated scales scores with a mean of 10 and SD of 3, and the SDQ which provides a cumulative raw score. All standard scores were converted to z-scores for analysis using the test means and SDs, while the SDQ raw score was converted to a z-score using the mean and SD of the sample population. Assessors were blinded to sepsis status, and any interventions offered post discharge that may affect neurodevelopment did not differ between sepsis groups.

### Data and statistical analyses

All statistical analyses were conducted using Stata (StataCorp, TX). Rates of sepsis between participants and non-participants were compared. Participant characteristics were described using means (standard deviations) or number (%), by sepsis status. Analysis included all participants with available data.

To address aim 1, the mean trajectories of each regional brain volume of interest from term-equivalent age to 13 years were described for those with and without neonatal sepsis, using linear mixed effects models. Sepsis status and age (3-level variable: 0, 7 and 13 years) were included as fixed effects, with a random intercept to allow for correlations between repeated observations within participants at the different time-points. Interaction terms were included to allow for the mean trajectories from 0 to 7 and 7 to 13 years to differ between the participants who did and did not have neonatal sepsis. All models were adjusted for sex, and additional analyses were adjusted for total brain volume to account for potential inter-subject variability in overall brain size.

To address aim 2, separate linear regressions were used to estimate mean differences in each brain volume and neurodevelopmental outcome measure at 13 years between sepsis and no sepsis groups. Models were fitted using generalised estimating equations to allow for clustering of multiple births within the same family and adjusted for sex, gestational age and birthweight SD score (see Table 1). Additional analyses were performed adding white matter injury (WMI) grade scored on term-equivalent scans^30^ and postnatal corticosteroid exposure as covariates. This additional adjustment was necessary because we did not have precise times of all events, particularly any that might lead to WMI and also sepsis, and hence it is unclear whether WMI and postnatal corticosteroid exposure are common causes of sepsis, brain volume and neurodevelopment, or if they lie on the causal pathway between sepsis and brain volume or neurodevelopment. If addition of WMI and postnatal corticosteroid exposure altered any conclusions, we intended to add interaction terms for sepsis with WMI, and for sepsis with postnatal corticosteroid exposure to test for evidence of different sepsis-volume or sepsis-outcome associations within WMI or postnatal corticosteroid exposure subgroups. Our causal assumptions for the relationships between variables are depicted in a Directed Acyclic Graph (Supplementary Fig. 1).

**Table 1:** Characteristics of the very preterm infants, by sepsis status.

	Neurodevelopmental data		Volumetric data	
	Sepsis, *n* = 63	No Sepsis, *n* = 114	Sepsis, *n* = 79	No Sepsis, *n* = 137
Gestational age - weeks, mean (SD)	26.7 (1.9)	27.8 (1.9)	26.9 (1.8)	27.8 (1.8)
Birthweight - g, mean (SD)	874 (218)	1008 (216)	866 (214)	1012 (211)
Birthweight SD score, mean (SD)^*a*^	−0.6 (0.9)	−0.5 (0.9)	−0.7 (0.9)	−0.5 (0.9)
Small for gestational age, *n* (%)	5 (8)	9 (8)	8 (10)	11 (8)
Multiple birth, *n* (%)	26 (41)	53 (46)	32 (41)	59 (43)
Male sex, *n* (%)	31 (49)	61 (54)	38 (48)	74 (54)
Postnatal corticosteroids, *n* (%)	12 (19)	5^*b*^ (4)	15 (19)	*6*^*c*^ (4)
Bronchopulmonary dysplasia, *n* (%)^*d*^	29 (46)	34 (30)	33 (42)	40 (29)
Cystic periventricular leukomalacia, *n* (%)^*e*^	2 (3)	5 (4)	2 (3)	7 (5)
Intraventricular haemorrhage grade 3 or 4, *n* (%)^*f*^	1 (2)	7 (6)	1 (1)	7 (5)
White matter injury, *n* (%)^*g*^				
None	26 (41)	53 (46)	32 ^*h*^ (41)	61^*c*^ (45)
Mild	26 (41)	42 (37)	30 ^*h*^ (38)	49^*c*^ (36)
Moderate	7 (11)	15 (13)	11 ^*h*^ (14)	20^*c*^ (15)
Severe	4 (6)	4 (4)	5 ^*h*^ (6)	6^*c*^ (4)
24-month MDI standard score, mean (SD)	82^*i*^ (19)	87^*b*^ (18)	80^*j*^ (21)	86^*k*^ (18)
7-year IQ, mean (SD)	96 ^*l*^ (13)	99 ^*m*^ (14)	95^*n*^ (13)	98^*o*^ (14)
Higher social risk, *n* (%)^*p*^	38^q^ (62)	60^*r*^ (56)	38^*q*^ (62)	60^*r*^ (56)

IQ= Intelligence Quotient, measured from The Wechsler Abbreviated Scale of Intelligence (WASI).^42^ *MDI* Mental Development Index, measured from Bailey Scales of Infant Development – 2nd Edition (BSID-II).^43^ *SD* standard deviation. ^a^Birthweight SD score was calculated relative to the British Growth Reference dataset (Cole et al. ^44^). Small for gestational age was defined as birthweight SD score <-2. ^b^*n* = 113. ^c^ *n* = 136. ^d^Bronchopulmonary dysplasia was defined as requirement for supplemental oxygen at 36 weeks. ^e^Recorded from cranial ultrasound. ^f^Recorded from cranial ultrasound, graded according to Papile et al.^45^ ^g^Graded on term-equivalent scans using Kidokoro et al.^30^ and classified as previously described.^6^ ^h^*n* = 78. ^i^*n* = 62. ^j^*n* = 77. ^k^*n* = 131. ^l^*n* = 59. ^m^*n* = 110. ^n^*n* = 64. ^o^*n* = 122. ^p^Calculated at 13 years using Treyvaud et al.^46^ and dichotomised around the median score of 2. ^q^*n* = 61. ^r^*n* = 107.

All estimates are presented with 95% confidence intervals and *p* values that were false discovery rate-corrected^31^ based on the number of brain regions (aims 1 and 2) or neurodevelopment outcomes (aim 2).

## Results

### Participant characteristics

**Fig. 1:**
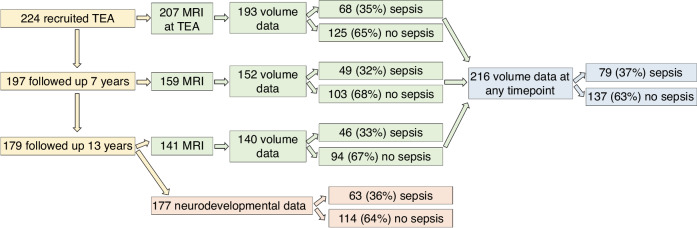
Flowchart of participants recruited and included in the study. TEA term-equivalent age (38–42 weeks’ gestation), MRI magnetic resonance imaging.

At age 7 years, corrected for prematurity, 197 (88%) VP children were followed up, and at 13 years’ corrected age, 179 (80%) VP children were followed up. Of those who returned at 13 years of age, 177 children had usable neurodevelopmental data, 63 (36%) with sepsis and 114 (64%) without sepsis. Following visual inspection of segmentation quality, usable volumetric MRI data were available for 193 infants at term-equivalent age (86% of original cohort); for 152 7-year-old children (67% of original cohort) and for 140 13-year-olds (63% of original cohort) (Fig. 1).

In all, 216 VP children had usable brain volumetric data at any time-point (*n* = 79 sepsis, *n* = 137 no sepsis), and all these children were included in the longitudinal analyses. The characteristics of the participants are summarised in Table 1. Infants with sepsis were born at lower gestational age and lower birthweight compared with infants who did not have sepsis and were more likely to have received postnatal corticosteroids. Additionally, for those who had a neurodevelopmental assessment at 13 years, those with sepsis were more likely to have had bronchopulmonary dysplasia. All other characteristics were similar between the two groups. The rates of sepsis between participants and non-participants were similar for both the volumetric data (37% [79/216] participants had sepsis, 13% [1/8] non-participants had sepsis) and neurodevelopmental data (36% [63/177] participants had sepsis, 38% [18/47] non-participants had sepsis). The main organisms causing sepsis were coagulase negative Staphylococci (80%), *Staphylococcus aureus* (15%), *Candida* (8%), gram negative organisms (*Klebsiella sp*., *Escherichia coli* and *Enterobacter sp*.; 4%), group B Streptococcus (3%) and enterococcal infection (1%).^6^ 10% of the sepsis group (*n* = 8) had NEC, where 4 had both sepsis and NEC and 4 had NEC only.

### Brain volumes from term-equivalent age to 13 years

**Fig. 2:**
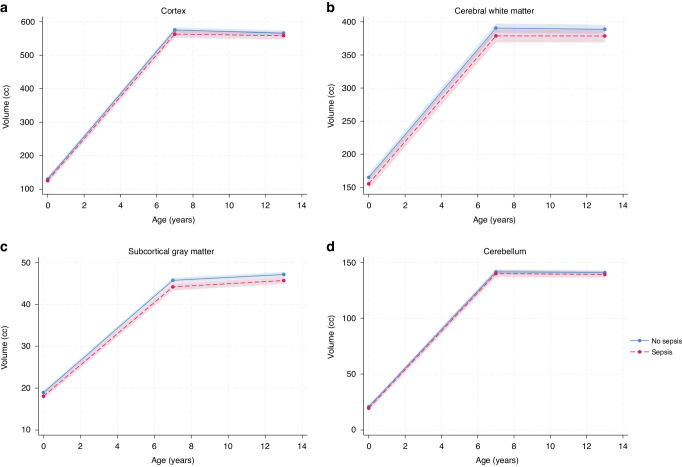
Growth trajectory of major brain tissues and regions between term-equivalent age, 7 years and 13 years of age for very preterm infants in the sepsis and no sepsis group. **a** cerebral cortex, **b** cerebral white matter, **c** subcortical grey matter, **d** cerebellum. Note: Red dashed lines represent the sepsis group and blue solid lines represent the no sepsis group; shading represents 95% confidence intervals; hemispheres are combined; subcortical grey matter is made up of thalamus, basal ganglia (caudate, putamen, pallidum, accumbens), hippocampus and amygdala; points at each age represent mean volume; models were adjusted for sex and mean volumes are averaged over sex. cc – cubic centimetres.

For those with and without neonatal sepsis, there was a rapid increase in brain volumes between infancy and 7 years which slowed by 13 years (Fig. 2). Volumetric trajectories between the groups were similar from term-equivalent age to 7 years of age (Supplementary Table 1a), and from 7 to 13 years of age for all brain regions (Supplementary Table 1b). Results were similar for trajectories after adjusting for total brain volume (Supplementary Table 1c and 1d).

### Brain volumes at 13 years

At 13 years of age, the neonatal sepsis group had similar regional brain volumes to those with no sepsis, adjusting for sex, gestational age and birthweight SD score (Supplementary Table 2). After additionally including WMI and postnatal corticosteroid exposure in the model, regional brain volumes were still similar between the sepsis groups (Supplementary Table 2).

### Neurodevelopmental outcomes at 13 years

**Fig. 3:**
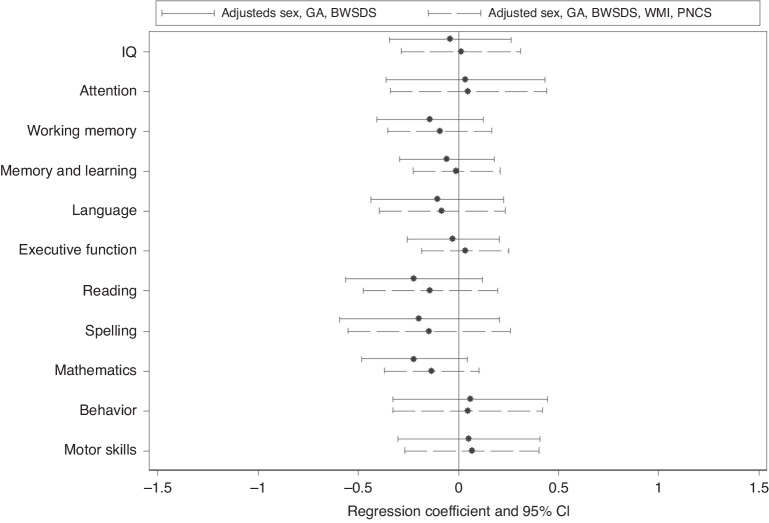
Mean differences in neurodevelopment outcomes between the sepsis and no sepsis groups. Note: Neurodevelopmental outcomes are presented as z-scores with 95% confidence intervals (CI) adjusted for sex, gestational age (GA) and birthweight standard deviation score (BWSDS) (solid line), and additionally adjusted for white matter injury (WMI) and postnatal corticosteroid exposure (PNCS) (dashed line); negative coefficients represent less favourable outcomes for the children who had perinatal sepsis and more favourable outcomes for those without sepsis, except for behaviour, where positive coefficients are less favourable for children with neonatal sepsis.

While point estimates supported a general trend for the sepsis group to perform worse than the no-sepsis group, especially for academics, 95% confidence intervals generally included the possibility of no sepsis group differences. Adjustment for WMI and postnatal corticosteroid exposure altered no conclusions, although the magnitude of group differences was reduced (Fig. 3, Supplementary Table 3).

## Discussion

There was little evidence that volumetric trajectories from term-equivalent age to 13 years of age differed with neonatal sepsis, or that neonatal sepsis in the VP infant was associated with regional brain volumetric alterations or adverse neurodevelopmental outcomes at13-years.

A unique aspect of this study is the serial MRI data from term-equivalent age, 7, and 13 years of age, enabling us to compare regional brain growth trajectories in VP children who did and did not have neonatal sepsis through childhood. Volumetric growth trajectories were similar between sepsis groups, with a rapid increase between infancy and 7 years which slowed by 13 years, which we have previously detailed in this group of VP and at term-born children.^12^

We found little evidence for differences in brain volume between those born VP who did or did not have neonatal sepsis after taking into account sex and immaturity (gestational age and birthweight SD score), indicating that perinatal factors other than sepsis contribute more to reduced brain volumes observed in 13 year olds born VP.^12^ There is a dearth of studies describing long-term effects of neonatal sepsis on regional brain volumes in those born VP. Even in infancy, the effects of sepsis on brain volumes are unclear, with some studies reporting reduced cerebral volumes,^13,14^ while others found no associations.^32,33^ Our current findings suggest there may not be a clinically important long-term adverse effect of sepsis per se on regional brain volumes. Future research would benefit from multi-modal imaging analyses to provide additional information on the neurological impact of neonatal sepsis than volumetry alone, including MR spectroscopy^34^ and diffusion techniques,^35^ which have been previously applied to examine the neurological impact of postnatal sepsis in VP infants.

We did not find strong evidence of functional neurodevelopmental consequences for neonatal sepsis in the VP group at 13 years of age. This suggests that previously documented negative effects of neonatal sepsis on neurodevelopmental outcomes observed in VP infants^5^ and 2-year-olds^6^ may weaken with increasing age,^7^ or that health, intervention, and education differences over the years may mask the effects. However, it is possible that our study was underpowered to identify meaningful differences between groups. We did observe trends of reduced standard scores for academics (reading, spelling and mathematics), working memory and language in the sepsis compared with no sepsis group, which may be clinically meaningful. Our previous meta-analysis on the long-term effects of neonatal sepsis found increased risk of neurodevelopmental disability in VP infants followed up beyond 18 months, but could not conclude on effects beyond 36 months, due to a paucity of studies.^7^ A few studies have reported greater adverse outcomes following sepsis in older children born preterm or very low birthweight, including motor problems, lower IQ, and memory and attention impairments in 6–9 year olds,^36^ worse general cognition, language, academic achievement, and executive function in 10 year olds,^37^ and an association with disability status in 12–15 year olds.^38^ However, these previous studies differed from ours in definition and timing of sepsis, study numbers and confounders used for analyses, and in the version or tests used for neurodevelopmental assessment, meaning it is difficult to directly compare findings. Even so, unlike the current study, these previous studies indicate ongoing neurodevelopmental problems following neonatal sepsis.

We previously reported that WMI mediated the association between postnatal sepsis and poor 2-year neurodevelopmental outcomes in this VP cohort.^6^ While the strength of the associations for all outcomes weakened slightly with adjustment for WMI in the current analysis, there was little evidence that neonatal WMI influenced the relationship between sepsis and neurodevelopmental outcome at 13 years. Other studies have documented adverse neurodevelopmental outcomes in VP children with sepsis related to white matter microstructural abnormalities^39^ and cerebral lesions.^40^ It would be anticipated that the WMI resulting from neonatal sepsis would remain impactful in childhood when higher processing functions can be tested. However, this was not found in our current study.

This study has several limitations. While we report a relatively high follow-up rate of 80%, loss to follow up is an inherent limitation in longitudinal studies. In addition, the small and uneven sample sizes between the sepsis groups further reduced study power. MR images were more likely to be excluded in individuals with brain pathology due to poor segmentation, which may have reduced the size of the differences between the sepsis groups. Variables in this study were subject to measurement error which may have led to bias in our estimates. In particular, data on suspected but not proven sepsis were not collected due to subjectivity in measuring suspected sepsis. Hence the no sepsis group may have included individuals with suspected but not proven sepsis, which may have diluted our results. Furthermore, we modelled brain growth linearly. While complex brain growth trajectories are likely to be best described by nonlinear models,^41^ they were not appropriate as our data were very highly clustered around only 3 time-points.

## Conclusion

This study provides insight into early adolescent outcomes of VP infants with neonatal sepsis. There was little difference in volumetric growth trajectories related to neonatal sepsis, and there was little evidence for reduced volumes in any region of the brain for VP adolescents with neonatal sepsis. While there was a trend for poorer functioning in the sepsis group that was potentially clinically meaningful, in particular for academics, the evidence for differences in neurodevelopmental outcomes at 13 years of age between sepsis groups was generally weak. Together these results provide little evidence that neonatal sepsis directly contributes to long-term altered brain structure and function in VP adolescents. An improved understanding of the brain architecture and long-term neurodevelopmental outcomes with high-quality prospective clinical cohorts will be needed to further confirm the current study findings.

## Supplementary information

Supplementary information

## Data Availability

The datasets generated during and analysed during the current study are not publicly available due to ethical restrictions but are available from the corresponding author on reasonable request and completion of a data sharing agreement.
