# Supplementary material for: Brain volume and neurodevelopment at 13 years following sepsis in very preterm infants
Source: Pediatr Res. 2024 Jul 13;97(2):744–50. doi: 10.1038/s41390-024-03407-w (PMC12015111; doi:10.1038/s41390-024-03407-w)
Supplement: Supplementary file 1 — Supplementary information [file 41390_2024_3407_MOESM1_ESM.pdf]

Supplementary Figure 1. Directed Acyclic Graph depicting the assumptions made for Aim 2. Note: It is unclear whether white matter injury and postnatal steroid exposure are common causes of sepsis, brain volume and neurodevelopment, or on the causal pathway between sepsis and brain volume or neurodevelopment. Therefore, analyses were conducted both with and without regression adjustment for white matter injury and postnatal steroid exposure.

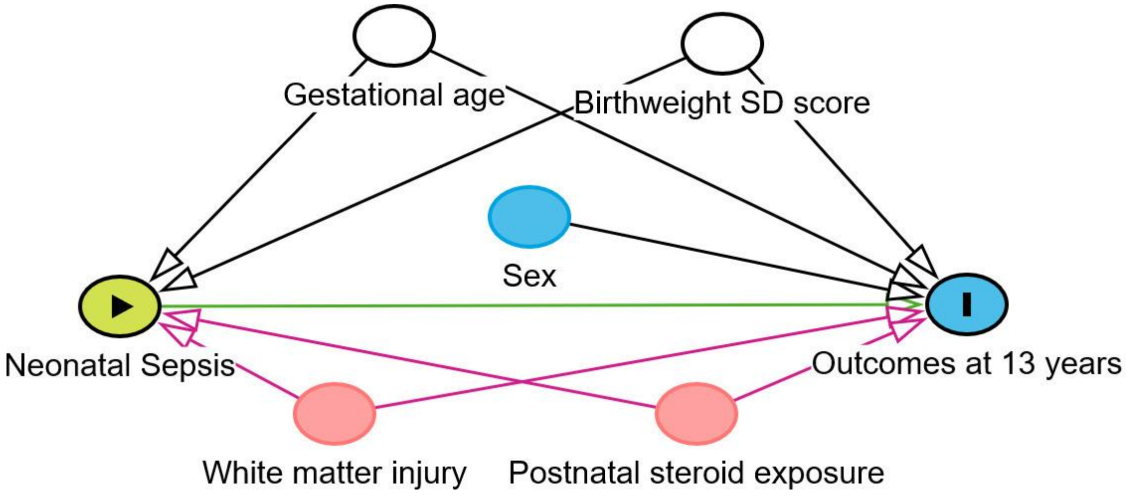

Supplementary Table 1a. Difference in mean brain volumes (cm^3) between term-equivalent and 7 years of age, for children who did and did not have neonatal sepsis, not adjusted for total brain volume.

	Sepsis			No sepsis			Interaction β	Interaction *p*
	β	95% CI lower	95% CI upper	β	95% CI lower	95% CI upper		
Intracranial	886	860	911	898	881	916	-12.5	0.99
Total brain tissue	825	802	848.1	832	816	848	-6.78	0.99
Extra-axial cerebrospinal fluid	53.5	41.7	65.3	55.5	47.3	63.7	-1.99	0.99
Cerebrospinal fluid	61.6	49.8	73.4	66.4	58.3	74.6	-4.79	0.99
Total cortical grey matter	439	427	451	446	438	455	-7.57	0.99
Left cortical grey matter	219	213	225	223	218	227	-3.34	0.99
Right cortical grey matter	219	213	225	224	219	228	-4.23	0.93
Left lateral ventricle	2.84	1.27	4.41	2.68	1.58	3.78	0.16	0.99

Right lateral ventricle	2.97	1.50	4.45	3.32	2.29	4.34	-0.34	0.99	
Third ventricle	0.42	0.33	0.51	0.44	0.38	0.51	-0.02	0.99	
Fourth ventricle	1.35	1.21	1.49	1.35	1.25	1.45	-0.01	0.99	
Left white matter	112	107	117	112	109	116	-0.20	0.99	
Right white matter	113	108	117	113	109	116	-0.27	0.99	
Total white matter	225	215	234	225	218.3	232	-0.46	0.99	
Corpus callosum	2.39	2.25	2.54	2.44	2.34	2.54	-0.05	0.99	
Brainstem	14.3	13.8	14.8	14.5	14.2	14.9	-0.25	0.99	
Left cerebellum	60.9	59.2	62.5	60.7	59.5	61.8	0.21	0.99	
Right cerebellum	59.6	58.0	61.3	60.3	59.1	61.5	-0.66	0.99	
Left thalamus	3.29	3.11	3.47	3.32	3.20	3.44	-0.03	0.99	
Left caudate	2.40	2.30	2.51	2.53	2.45	2.60	-0.12	0.76	
Left putamen	3.18	3.04	3.32	3.25	3.15	3.35	-0.07	0.99	
Left pallidum	1.08	1.03	1.14	1.18	1.14	1.22	-0.10	0.21	
Left hippocampus	1.77	1.69	1.84	1.80	1.75	1.85	-0.03	0.99	
Left amygdala	0.81	0.77	0.86	0.83	0.80	0.86	-0.02	0.99	
Left accumbens	0.35	0.33	0.38	0.32	0.30	0.34	0.03	0.61	
Right thalamus	3.11	2.96	3.26	3.15	3.05	3.25	-0.04	0.99	
Right caudate	2.50	2.39	2.61	2.61	2.54	2.69	-0.12	0.76	
Right putamen	3.39	3.27	3.51	3.43	3.35	3.52	-0.05	0.99	
Right pallidum	0.98	0.92	1.03	1.08	1.04	1.12	-0.10	0.21	
Right hippocampus	1.95	1.88	2.02	1.95	1.91	2.00	0.00	0.99	
Right amygdala	1.04	0.99	1.08	1.03	1.00	1.06	0.01	0.99	

Right accumbens	0.42	0.39	0.44	0.41	0.40	0.43	0.00	0.99
Left banks superior temporal sulcus	2.55	2.39	2.71	2.53	2.42	2.65	0.01	0.99
Left caudal anterior cingulate	1.19	1.05	1.33	1.25	1.16	1.35	-0.07	0.99
Left caudal middle frontal	6.58	6.23	6.93	6.45	6.20	6.70	0.13	0.99
Left cuneus	2.03	1.85	2.20	2.05	1.93	2.18	-0.03	0.99
Left entorhinal	1.82	1.64	1.99	1.83	1.71	1.96	-0.02	0.99
Left fusiform	8.60	8.26	8.94	8.95	8.72	9.19	-0.35	0.76
Left inferior parietal	11.8	11.2	12.3	11.8	11.4	12.2	-0.07	0.99
Left inferior temporal	11.9	11.4	12.4	12.1	11.7	12.5	-0.21	0.99
Left isthmus cingulate	2.38	2.24	2.53	2.38	2.28	2.48	0.00	1.00
Left lateral occipital	11.4	10.8	12.0	11.5	11.1	11.9	-0.14	0.99
Left lateral orbital frontal	6.34	6.08	6.61	6.48	6.29	6.67	-0.14	0.99
Left lingual	5.36	5.07	5.66	5.74	5.53	5.95	-0.37	0.61
Left medial orbital frontal	4.57	4.39	4.76	4.78	4.65	4.90	-0.20	0.76
Left middle temporal	10.6	10.2	11.1	10.7	10.4	11.0	-0.06	0.99
Left parahippocampal	1.86	1.73	1.99	1.97	1.88	2.07	-0.11	0.76
Left paracentral	3.06	2.90	3.22	3.04	2.93	3.16	0.01	0.99
Left pars opercularis	4.59	4.35	4.84	4.58	4.41	4.75	0.02	0.99
Left pars orbitalis	2.34	2.22	2.45	2.33	2.24	2.41	0.01	0.99
Left pars triangularis	3.62	3.45	3.79	3.68	3.56	3.80	-0.06	0.99
Left pericalcarine	0.26	0.13	0.40	0.27	0.17	0.36	0.00	0.99
Left postcentral	6.93	6.48	7.37	6.86	6.55	7.17	0.07	0.99
Left posterior cingulate	2.68	2.52	2.83	2.76	2.65	2.87	-0.08	0.99

Left precentral	11.3	10.8	11.8	11.6	11.3	12.0	-0.33	0.93
Left precuneus	10.1	9.66	10.5	10.7	10.4	10.9	-0.59	0.57
Left rostral anterior cingulate	2.43	2.27	2.58	2.45	2.34	2.56	-0.02	0.99
Left rostral middle frontal	17.6	16.8	18.3	17.8	17.3	18.4	-0.29	0.99
Left superior frontal	22.5	21.6	23.3	22.3	21.8	22.9	0.13	0.99
Left superior parietal	12.8	12.2	13.5	13.2	12.8	13.7	-0.43	0.98
Left superior temporal	12.6	12.0	13.1	12.6	12.3	13.0	-0.10	0.99
Left supramarginal	10.9	10.3	11.5	11.5	11.0	11.9	-0.52	0.76
Left frontal pole	1.19	1.13	1.26	1.18	1.13	1.22	0.02	0.99
Left temporal pole	2.13	2.00	2.26	2.07	1.98	2.17	0.06	0.99
Left transverse temporal	0.82	0.76	0.88	0.81	0.76	0.85	0.01	0.99
Left insula	6.11	5.87	6.35	6.34	6.17	6.51	-0.23	0.76
Right banks superior temporal sulcus	2.01	1.89	2.13	2.15	2.07	2.24	-0.14	0.76
Right caudal anterior cingulate	1.21	1.05	1.38	1.31	1.19	1.42	-0.09	0.99
Right caudal middle frontal	6.11	5.75	6.47	6.03	5.77	6.28	0.08	0.99
Right cuneus	2.46	2.27	2.65	2.35	2.22	2.48	0.11	0.99
Right entorhinal	1.84	1.67	2.01	1.82	1.70	1.94	0.02	0.99
Right fusiform	8.08	7.72	8.44	8.33	8.08	8.59	-0.26	0.93
Right inferior parietal	14.6	13.9	15.3	15.2	14.7	15.7	-0.62	0.76
Right inferior temporal	12.3	11.7	12.9	12.3	11.9	12.8	-0.08	0.99
Right isthmus cingulate	1.86	1.73	1.99	2.04	1.95	2.14	-0.18	0.61
Right lateral occipital	12.3	11.7	12.9	12.0	11.5	12.4	0.34	0.99
Right lateral orbital frontal	6.23	5.97	6.49	6.36	6.17	6.54	-0.13	0.99

Right lingual	5.79	5.46	6.12	6.03	5.80	6.26	-0.24	0.93
Right medial orbital frontal	5.29	5.10	5.48	5.36	5.22	5.49	-0.07	0.99
Right middle temporal	10.8	10.4	11.3	11.2	10.9	11.6	-0.43	0.76
Right parahippocampal	1.55	1.46	1.64	1.67	1.61	1.74	-0.13	0.57
Right paracentral	3.59	3.42	3.75	3.55	3.43	3.66	0.04	0.99
Right pars opercularis	3.55	3.35	3.76	3.62	3.48	3.77	-0.07	0.99
Right pars orbitalis	2.93	2.80	3.05	2.99	2.90	3.08	-0.06	0.99
Right pars triangularis	4.22	4.00	4.45	4.36	4.20	4.53	-0.14	0.99
Right pericalcarine	0.41	0.27	0.56	0.41	0.31	0.51	0.00	0.99
Right postcentral	6.44	6.00	6.88	6.87	6.56	7.18	-0.43	0.76
Right posterior cingulate	2.49	2.34	2.65	2.63	2.52	2.74	-0.14	0.76
Right precentral	11.5	11.0	12.0	11.6	11.2	11.9	-0.10	0.99
Right precuneus	10.6	10.2	11.0	11.0	10.7	11.3	-0.40	0.76
Right rostral anterior cingulate	1.53	1.41	1.65	1.60	1.52	1.69	-0.07	0.99
Right rostral middle frontal	18.0	17.2	18.7	18.3	17.8	18.8	-0.32	0.99
Right superior frontal	21.5	20.6	22.3	21.2	20.7	21.8	0.22	0.99
Right superior parietal	12.3	11.6	12.9	12.9	12.5	13.4	-0.62	0.76
Right superior temporal	11.6	11.1	12.1	11.8	11.4	12.1	-0.15	0.99
Right supramarginal	9.52	9.04	10.0	9.88	9.54	10.2	-0.35	0.93
Right frontal pole	1.33	1.25	1.41	1.33	1.28	1.39	0.00	0.99
Right temporal pole	2.14	2.01	2.27	2.26	2.16	2.35	-0.12	0.76
Right transverse temporal	0.57	0.53	0.62	0.56	0.53	0.59	0.02	0.99
Right insula	5.97	5.74	6.21	6.15	5.98	6.31	-0.17	0.93

Regression coefficients (β) and p-values (false discovery rate corrected) were derived from linear mixed effects models adjusted for sex. Positive β indicates brain volumes increased over time; negative β indicates brain volumes reduced over time. Positive interaction β indicate brain volumes increased more over time in those with neonatal sepsis compared with those without neonatal sepsis; negative interaction β indicate brain volumes increased less over time in those with neonatal sepsis compared with those without neonatal sepsis. N/A= not applicable, CI= confidence interval.

Supplementary Table 1b. Difference in mean brain volumes (cm^3) between 7 and 13 years of age, for children who did and did not have neonatal sepsis, not adjusted for total brain volume.

	Sepsis			No sepsis			Interaction β	Interaction *p*
	β	95% CI lower	95% CI upper	β	95% CI lower	95% CI upper		
Intracranial	104	75.9	132	93.2	74.0	112	10.8	0.99
Total brain tissue	49.6	24.7	74.5	43.9	26.8	61.0	5.70	0.99
Extra-axial cerebrospinal fluid	51.4	38.7	64.1	49.3	40.6	58.0	2.11	0.99
Cerebrospinal fluid	54.2	41.6	66.9	49.5	40.8	58.2	4.80	0.99
Total cortical grey matter	-4.71	-17.9	8.47	-9.26	-18.5	-0.04	4.54	0.99
Left cortical grey matter	-2.85	-9.52	3.81	-4.48	-9.14	0.18	1.63	0.99
Right cortical grey matter	-1.86	-8.42	4.70	-4.77	-9.36	-0.19	2.91	0.99
Left lateral ventricle	1.35	-0.33	3.03	1.08	-0.06	2.23	0.27	0.99
Right lateral ventricle	1.53	-0.05	3.10	1.07	-0.01	2.15	0.46	0.99
Third ventricle	0.08	-0.02	0.18	0.02	-0.05	0.09	0.06	0.99
Fourth ventricle	0.20	0.05	0.36	0.16	0.06	0.27	0.04	0.99
Left white matter	-0.04	-5.45	5.38	-0.76	-4.54	3.02	0.72	0.99
Right white matter	-0.13	-5.50	5.25	-1.05	-4.80	2.70	0.92	0.99
Total white matter	-0.16	-10.9	10.6	-1.81	-9.31	5.70	1.64	0.99
Corpus callosum	0.09	-0.07	0.25	0.12	0.01	0.23	-0.03	0.99
Brainstem	2.52	1.96	3.07	2.52	2.13	2.90	0.00	1.00

Left cerebellum	-1.00	-2.92	0.93	-0.81	-2.13	0.52	-0.19	0.99
Right cerebellum	0.19	-1.77	2.14	0.22	-1.13	1.57	-0.04	0.99
Left thalamus	0.57	0.38	0.77	0.47	0.33	0.60	0.11	0.99
Left caudate	-0.06	-0.18	0.06	-0.08	-0.16	0.00	0.02	0.99
Left putamen	0.11	-0.05	0.26	0.09	-0.01	0.20	0.01	0.99
Left pallidum	0.17	0.11	0.23	0.16	0.12	0.20	0.01	0.99
Left hippocampus	0.07	-0.01	0.15	0.09	0.04	0.14	-0.02	0.99
Left amygdala	0.11	0.06	0.15	0.10	0.06	0.13	0.01	0.99
Left accumbens	-0.03	-0.06	-0.01	0.00	-0.02	0.02	-0.03	0.95
Right thalamus	0.37	0.21	0.53	0.32	0.21	0.43	0.05	0.99
Right caudate	-0.05	-0.17	0.06	-0.04	-0.12	0.04	-0.02	0.99
Right putamen	0.04	-0.09	0.17	0.09	0.00	0.18	-0.05	0.99
Right pallidum	0.15	0.09	0.21	0.09	0.05	0.14	0.06	0.99
Right hippocampus	0.01	-0.07	0.09	0.05	-0.01	0.10	-0.04	0.99
Right amygdala	0.06	0.01	0.10	0.07	0.04	0.10	-0.01	0.99
Right accumbens	0.01	-0.01	0.04	0.00	-0.02	0.02	0.01	0.99
Left banks superior temporal sulcus	-0.30	-0.48	-0.13	-0.20	-0.32	-0.08	-0.10	0.99
Left caudal anterior cingulate	0.17	0.02	0.33	0.13	0.03	0.24	0.04	0.99
Left caudal middle frontal	0.21	-0.18	0.60	0.12	-0.15	0.39	0.09	0.99
Left cuneus	-0.16	-0.34	0.03	-0.08	-0.21	0.05	-0.08	0.99
Left entorhinal	0.20	0.00	0.39	-0.04	-0.18	0.10	0.24	0.99
Left fusiform	-0.11	-0.48	0.27	-0.11	-0.36	0.15	0.00	1.00
Left inferior parietal	-0.96	-1.58	-0.35	-0.95	-1.37	-0.52	-0.02	0.99

Left inferior temporal	-0.23	-0.80	0.34	-0.18	-0.57	0.21	-0.05	0.99
Left isthmus cingulate	-0.10	-0.26	0.05	-0.08	-0.18	0.03	-0.03	0.99
Left lateral occipital	-0.73	-1.34	-0.12	-0.96	-1.38	-0.54	0.23	0.99
Left lateral orbital frontal	0.29	0.00	0.58	-0.01	-0.20	0.19	0.29	0.99
Left lingual	-0.11	-0.44	0.21	-0.07	-0.29	0.16	-0.05	0.99
Left medial orbital frontal	0.08	-0.12	0.27	-0.25	-0.39	-0.12	0.33	0.70
Left middle temporal	-0.10	-0.56	0.37	-0.12	-0.44	0.20	0.02	0.99
Left parahippocampal	0.04	-0.11	0.18	0.07	-0.03	0.17	-0.03	0.99
Left paracentral	-0.10	-0.28	0.08	-0.15	-0.27	-0.03	0.05	0.99
Left pars opercularis	0.07	-0.19	0.33	-0.02	-0.20	0.16	0.09	0.99
Left pars orbitalis	-0.10	-0.23	0.02	-0.19	-0.28	-0.10	0.08	0.99
Left pars triangularis	-0.06	-0.24	0.12	-0.13	-0.25	0.00	0.07	0.99
Left pericalcarine	0.21	0.05	0.36	0.28	0.17	0.39	-0.07	0.99
Left postcentral	0.05	-0.46	0.57	-0.12	-0.48	0.23	0.18	0.99
Left posterior cingulate	-0.03	-0.21	0.14	0.03	-0.09	0.15	-0.07	0.99
Left precentral	0.60	0.08	1.12	0.44	0.08	0.80	0.16	0.99
Left precuneus	-0.44	-0.88	0.00	-0.89	-1.19	-0.59	0.45	0.99
Left rostral anterior cingulate	0.18	0.02	0.35	0.21	0.09	0.32	-0.02	0.99
Left rostral middle frontal	0.32	-0.47	1.11	-0.28	-0.82	0.27	0.60	0.99
Left superior frontal	0.15	-0.73	1.03	-0.01	-0.62	0.59	0.16	0.99
Left superior parietal	-1.32	-2.02	-0.62	-1.09	-1.58	-0.61	-0.23	0.99
Left superior temporal	-0.51	-1.09	0.07	-0.41	-0.81	0.00	-0.10	0.99
Left supramarginal	-0.59	-1.26	0.09	-0.45	-0.92	0.01	-0.13	0.99

Left frontal pole	-0.23	-0.30	-0.16	-0.20	-0.25	-0.15	-0.03	0.99
Left temporal pole	-0.10	-0.25	0.04	-0.16	-0.26	-0.06	0.05	0.99
Left transverse temporal	-0.06	-0.13	0.01	-0.04	-0.09	0.00	-0.02	0.99
Left insula	-0.06	-0.32	0.21	-0.18	-0.36	0.00	0.13	0.99
Right banks superior temporal sulcus	-0.17	-0.30	-0.04	-0.20	-0.29	-0.11	0.03	0.99
Right caudal anterior cingulate	0.26	0.09	0.43	0.12	0.00	0.24	0.14	0.99
Right caudal middle frontal	0.30	-0.10	0.69	0.18	-0.09	0.45	0.12	0.99
Right cuneus	-0.20	-0.40	0.01	-0.15	-0.29	-0.01	-0.05	0.99
Right entorhinal	0.19	0.00	0.38	0.09	-0.04	0.22	0.10	0.99
Right fusiform	-0.13	-0.52	0.27	-0.09	-0.37	0.18	-0.03	0.99
Right inferior parietal	-0.35	-1.11	0.41	-0.88	-1.40	-0.36	0.53	0.99
Right inferior temporal	-0.03	-0.69	0.63	-0.06	-0.51	0.40	0.03	0.99
Right isthmus cingulate	-0.04	-0.19	0.10	-0.18	-0.28	-0.07	0.13	0.99
Right lateral occipital	-1.28	-1.96	-0.59	-1.01	-1.48	-0.53	-0.27	0.99
Right lateral orbital frontal	-0.10	-0.38	0.18	-0.23	-0.43	-0.04	0.13	0.99
Right lingual	-0.32	-0.67	0.04	-0.44	-0.68	-0.19	0.12	0.99
Right medial orbital frontal	-0.28	-0.49	-0.08	-0.23	-0.37	-0.09	-0.05	0.99
Right middle temporal	-0.19	-0.67	0.29	-0.17	-0.51	0.16	-0.01	0.99
Right parahippocampal	0.06	-0.04	0.15	0.05	-0.01	0.12	0.01	0.99
Right paracentral	-0.19	-0.37	0.00	-0.05	-0.18	0.08	-0.14	0.99
Right pars opercularis	0.09	-0.14	0.31	0.03	-0.13	0.18	0.06	0.99
Right pars orbitalis	0.03	-0.10	0.17	-0.17	-0.27	-0.08	0.21	0.80
Right pars triangularis	-0.01	-0.25	0.24	-0.14	-0.31	0.02	0.14	0.99

Right pericalcarine	0.29	0.13	0.45	0.41	0.31	0.52	-0.13	0.99
Right postcentral	-0.03	-0.52	0.45	-0.27	-0.60	0.06	0.24	0.99
Right posterior cingulate	0.14	-0.03	0.31	0.04	-0.07	0.16	0.10	0.99
Right precentral	0.76	0.22	1.31	0.78	0.40	1.16	-0.01	0.99
Right precuneus	-0.57	-1.03	-0.11	-0.99	-1.31	-0.67	0.42	0.99
Right rostral anterior cingulate	0.15	0.02	0.28	0.09	0.00	0.18	0.06	0.99
Right rostral middle frontal	-0.02	-0.87	0.83	-0.47	-1.06	0.12	0.45	0.99
Right superior frontal	0.22	-0.66	1.10	0.39	-0.21	1.00	-0.18	0.99
Right superior parietal	-0.61	-1.31	0.09	-1.17	-1.66	-0.69	0.56	0.99
Right superior temporal	-0.37	-0.91	0.16	-0.43	-0.80	-0.07	0.06	0.99
Right supramarginal	-0.11	-0.63	0.42	-0.35	-0.71	0.01	0.24	0.99
Right frontal pole	-0.25	-0.33	-0.16	-0.26	-0.32	-0.20	0.01	0.99
Right temporal pole	0.00	-0.14	0.14	-0.20	-0.30	-0.10	0.20	0.80
Right transverse temporal	-0.03	-0.08	0.02	-0.04	-0.08	-0.01	0.01	0.99
Right insula	0.20	-0.05	0.45	0.17	0.00	0.34	0.03	0.99

Regression coefficients (β) and p-values (false discovery rate corrected) were derived from linear mixed effects models adjusted for sex. Positive β indicate brain volumes increased over time; negative β indicate brain volumes reduced over time. Positive interaction β indicate brain volumes increased more over time in those with neonatal sepsis compared with those without neonatal sepsis; negative interaction β indicate brain volumes increased less over time in those with neonatal sepsis compared with those without neonatal sepsis. N/A= not applicable, CI= confidence interval.

Supplementary Table 1c. Difference in mean brain volumes (cm^3) between term-equivalent and 7 years of age, for children who did and did not have neonatal sepsis, adjusted for total brain volume.

	Sepsis			No sepsis			Interaction β	Interaction *p*
	β	95% CI lower	95% CI upper	β	95% CI lower	95% CI upper		
Extra-axial cerebrospinal fluid	19.9	-14.0	53.9	21.3	-12.1	54.6	-1.35	0.99

Cerebrospinal fluid	45.8	8.06	83.6	50.5	13.5	87.5	-4.66	0.99
Total cortical grey matter	90.2	72.0	109	95.1	77.4	113	-4.83	0.97
Left cortical grey matter	42.5	33.3	51.7	44.3	35.3	53.2	-1.76	0.99
Right cortical grey matter	47.8	38.5	57.1	50.9	41.8	59.9	-3.07	0.97
Left lateral ventricle	-0.39	-5.46	4.68	-0.64	-5.66	4.37	0.25	0.99
Right lateral ventricle	0.49	-4.35	5.32	0.75	-4.03	5.54	-0.27	0.99
Third ventricle	0.40	0.10	0.69	0.41	0.12	0.71	-0.01	0.99
Fourth ventricle	1.15	0.72	1.58	1.15	0.72	1.58	0.00	0.99
Left white matter	-43.2	-51.3	-35.2	-43.8	-51.6	-36.0	0.57	0.99
Right white matter	-36.7	-45.0	-28.4	-37.0	-45.1	-28.9	0.25	0.99
Total white matter	-80.3	-96.5	-64.2	-81.1	-96.7	-65.5	0.75	0.99
Corpus callosum	0.27	-0.10	0.64	0.28	-0.08	0.65	-0.01	0.99
Brainstem	3.42	2.20	4.64	3.44	2.23	4.65	-0.02	0.99
Left cerebellum	27.6	23.3	31.8	26.7	22.5	30.9	0.88	0.97
Right cerebellum	26.0	21.7	30.3	26.0	21.7	30.2	0.01	0.99
Left thalamus	-0.80	-1.25	-0.35	-0.85	-1.30	-0.41	0.05	0.99
Left caudate	0.31	0.00	0.63	0.39	0.08	0.70	-0.08	0.97
Left putamen	1.01	0.60	1.42	1.05	0.65	1.45	-0.04	0.99
Left pallidum	0.18	0.03	0.32	0.27	0.13	0.41	-0.09	0.35
Left hippocampus	0.40	0.21	0.60	0.40	0.21	0.60	0.00	0.99
Left amygdala	0.13	0.01	0.24	0.12	0.01	0.24	0.00	0.99
Left accumbens	0.09	0.02	0.16	0.05	-0.02	0.12	0.04	0.42
Right thalamus	-0.70	-1.11	-0.29	-0.73	-1.13	-0.32	0.03	0.99

Right caudate	0.37	0.05	0.69	0.43	0.11	0.74	-0.06	0.99
Right putamen	1.13	0.79	1.47	1.14	0.81	1.47	-0.01	0.99
Right pallidum	0.05	-0.10	0.20	0.14	-0.01	0.28	-0.09	0.35
Right hippocampus	0.68	0.47	0.88	0.65	0.45	0.85	0.03	0.99
Right amygdala	0.24	0.13	0.36	0.22	0.10	0.33	0.02	0.97
Right accumbens	0.09	0.03	0.15	0.08	0.02	0.14	0.01	0.99
Left banks superior temporal sulcus	0.47	0.07	0.88	0.42	0.03	0.81	0.06	0.99
Left caudal anterior cingulate	-0.61	-1.02	-0.21	-0.57	-0.96	-0.17	-0.05	0.99
Left caudal middle frontal	2.70	1.69	3.70	2.50	1.51	3.48	0.20	0.99
Left cuneus	0.20	-0.27	0.67	0.21	-0.25	0.67	-0.01	0.99
Left entorhinal	1.16	0.69	1.64	1.16	0.70	1.62	0.01	0.99
Left fusiform	2.19	1.37	3.01	2.43	1.62	3.24	-0.24	0.97
Left inferior parietal	1.55	0.16	2.93	1.46	0.10	2.83	0.08	0.99
Left inferior temporal	3.04	1.77	4.30	3.07	1.83	4.32	-0.04	0.99
Left isthmus cingulate	0.08	-0.28	0.44	0.04	-0.31	0.39	0.04	0.99
Left lateral occipital	1.44	0.08	2.80	1.40	0.06	2.74	0.04	0.99
Left lateral orbital frontal	0.15	-0.44	0.74	0.18	-0.40	0.75	-0.03	0.99
Left lingual	2.33	1.48	3.18	2.65	1.82	3.49	-0.32	0.97
Left medial orbital frontal	0.92	0.51	1.33	1.08	0.68	1.48	-0.16	0.97
Left middle temporal	1.86	0.89	2.83	1.79	0.84	2.75	0.07	0.99
Left parahippocampal	0.72	0.36	1.08	0.82	0.46	1.17	-0.10	0.97
Left paracentral	1.19	0.74	1.63	1.14	0.71	1.58	0.04	0.99
Left pars opercularis	1.76	1.10	2.43	1.68	1.03	2.34	0.08	0.99

Left pars orbitalis	0.84	0.52	1.16	0.81	0.50	1.12	0.04	0.99
Left pars triangularis	1.60	1.12	2.08	1.62	1.15	2.09	-0.02	0.99
Left pericalcarine	-0.94	-1.36	-0.51	-0.97	-1.39	-0.55	0.03	0.99
Left postcentral	-1.65	-2.73	-0.58	-1.90	-2.95	-0.84	0.24	0.97
Left posterior cingulate	-0.12	-0.51	0.27	-0.07	-0.46	0.31	-0.05	0.99
Left precentral	2.32	1.16	3.47	2.47	1.33	3.61	-0.15	0.99
Left precuneus	2.55	1.60	3.49	3.05	2.12	3.98	-0.51	0.42
Left rostral anterior cingulate	-0.11	-0.50	0.28	-0.12	-0.50	0.27	0.01	0.99
Left rostral middle frontal	3.83	2.17	5.49	3.93	2.32	5.55	-0.10	0.99
Left superior frontal	6.62	4.67	8.57	6.24	4.33	8.14	0.38	0.99
Left superior parietal	2.63	1.11	4.15	2.97	1.49	4.45	-0.34	0.99
Left superior temporal	1.84	0.65	3.03	1.83	0.66	2.99	0.01	0.99
Left supramarginal	1.45	-0.07	2.97	1.85	0.36	3.34	-0.40	0.97
Left frontal pole	0.82	0.65	0.98	0.79	0.63	0.95	0.03	0.99
Left temporal pole	0.98	0.66	1.29	0.92	0.61	1.22	0.06	0.99
Left transverse temporal	-0.23	-0.42	-0.04	-0.25	-0.44	-0.06	0.02	0.99
Left insula	1.12	0.55	1.68	1.27	0.72	1.82	-0.15	0.97
Right banks superior temporal sulcus	0.72	0.40	1.05	0.83	0.52	1.15	-0.11	0.97
Right caudal anterior cingulate	-0.80	-1.27	-0.33	-0.74	-1.21	-0.28	-0.05	0.99
Right caudal middle frontal	2.14	1.12	3.15	1.98	0.98	2.97	0.16	0.99
Right cuneus	-0.02	-0.50	0.46	-0.15	-0.62	0.32	0.13	0.97
Right entorhinal	0.71	0.25	1.18	0.67	0.22	1.13	0.04	0.99
Right fusiform	1.57	0.70	2.44	1.77	0.91	2.62	-0.20	0.97

Right inferior parietal	3.91	2.15	5.66	4.34	2.61	6.06	-0.43	0.97
Right inferior temporal	2.75	1.29	4.22	2.72	1.28	4.15	0.04	0.99
Right isthmus cingulate	-0.18	-0.52	0.16	-0.05	-0.38	0.29	-0.14	0.97
Right lateral occipital	2.83	1.26	4.40	2.35	0.81	3.89	0.48	0.97
Right lateral orbital frontal	0.31	-0.27	0.90	0.33	-0.25	0.90	-0.01	0.99
Right lingual	1.44	0.52	2.36	1.59	0.69	2.50	-0.15	0.99
Right medial orbital frontal	1.25	0.84	1.65	1.25	0.85	1.65	0.00	0.99
Right middle temporal	2.74	1.72	3.77	3.04	2.03	4.05	-0.30	0.97
Right parahippocampal	0.73	0.49	0.97	0.85	0.61	1.08	-0.12	0.53
Right paracentral	1.62	1.14	2.10	1.55	1.07	2.02	0.07	0.99
Right pars opercularis	0.88	0.33	1.43	0.89	0.36	1.43	-0.01	0.99
Right pars orbitalis	1.17	0.85	1.50	1.22	0.91	1.54	-0.05	0.99
Right pars triangularis	1.61	1.06	2.16	1.69	1.16	2.23	-0.08	0.99
Right pericalcarine	-1.41	-1.88	-0.94	-1.44	-1.90	-0.98	0.03	0.99
Right postcentral	-1.77	-2.85	-0.68	-1.43	-2.49	-0.37	-0.34	0.97
Right posterior cingulate	0.22	-0.20	0.63	0.31	-0.10	0.72	-0.10	0.97
Right precentral	2.98	1.81	4.15	2.96	1.82	4.10	0.02	0.99
Right precuneus	2.17	1.27	3.07	2.47	1.59	3.34	-0.30	0.97
Right rostral anterior cingulate	0.25	-0.10	0.60	0.31	-0.04	0.65	-0.05	0.99
Right rostral middle frontal	4.88	3.00	6.77	5.03	3.19	6.88	-0.15	0.99
Right superior frontal	5.52	3.57	7.46	5.11	3.22	7.00	0.41	0.99
Right superior parietal	2.64	1.26	4.01	3.17	1.83	4.50	-0.53	0.97
Right superior temporal	2.76	1.58	3.93	2.81	1.66	3.97	-0.05	0.99

Right supramarginal	0.90	-0.27	2.08	1.12	-0.03	2.27	-0.22	0.99
Right frontal pole	1.07	0.87	1.27	1.06	0.87	1.25	0.01	0.99
Right temporal pole	0.93	0.61	1.24	1.03	0.72	1.33	-0.10	0.97
Right transverse temporal	-0.13	-0.26	0.01	-0.16	-0.29	-0.02	0.03	0.97
Right insula	0.97	0.46	1.49	1.10	0.59	1.60	-0.12	0.97

Regression coefficients (β) and p-values (false discovery rate corrected) were derived from linear mixed effects models adjusted for sex. Positive β indicate brain volumes increased over time; negative β indicate brain volumes reduced over time. Positive interaction β indicate brain volumes increased more over time in those with neonatal sepsis compared with those without neonatal sepsis; negative interaction β indicate brain volumes increased less over time in those with neonatal sepsis compared with those without neonatal sepsis. N/A= not applicable, CI= confidence interval.

Supplementary Table 1d. Difference in mean brain volumes (cm^3) between 7 and 13 years of age, for children who did and did not have neonatal sepsis, adjusted for total brain volume.

	Sepsis			No sepsis			Interaction β	Interaction *p*
	β	95% CI lower	95% CI upper	β	95% CI lower	95% CI upper		
Extra-axial cerebrospinal fluid	48.9	36.1	61.8	47.5	38.6	56.4	1.45	0.99
Cerebrospinal fluid	52.5	39.6	65.3	47.9	38.9	56.8	4.63	0.99
Total cortical grey matter	-24.9	-32.6	-17.2	-27.9	-33.4	-22.5	3.06	0.99
Left cortical grey matter	-13.1	-17.0	-9.17	-13.9	-16.7	-11.1	0.82	0.99
Right cortical grey matter	-11.8	-15.7	-7.91	-14.0	-16.8	-11.3	2.23	0.99
Left lateral ventricle	1.07	-0.64	2.78	0.91	-0.25	2.08	0.16	0.99
Right lateral ventricle	1.29	-0.32	2.89	0.92	-0.17	2.02	0.36	0.99
Third ventricle	0.07	-0.04	0.17	0.01	-0.06	0.08	0.06	0.99
Fourth ventricle	0.18	0.02	0.34	0.15	0.04	0.26	0.03	0.99
Left white matter	-9.28	-12.7	-5.89	-9.03	-11.4	-6.63	-0.25	0.99
Right white matter	-9.13	-12.5	-5.75	-9.17	-11.6	-6.79	0.04	0.99

Total white matter	-18.4	-25.2	-11.7	-18.2	-23.0	-13.5	-0.17	0.99
Corpus callosum	-0.04	-0.18	0.11	0.03	-0.07	0.13	-0.06	0.99
Brainstem	1.85	1.42	2.28	2.06	1.77	2.36	-0.22	0.99
Left cerebellum	-3.10	-4.67	-1.52	-2.28	-3.37	-1.20	-0.81	0.99
Right cerebellum	-1.93	-3.55	-0.31	-1.27	-2.38	-0.15	-0.66	0.99
Left thalamus	0.32	0.15	0.49	0.29	0.17	0.40	0.03	0.99
Left caudate	-0.19	-0.30	-0.09	-0.17	-0.24	-0.10	-0.02	0.99
Left putamen	-0.01	-0.16	0.13	0.00	-0.10	0.10	-0.01	0.99
Left pallidum	0.12	0.06	0.17	0.12	0.08	0.16	0.00	0.99
Left hippocampus	-0.02	-0.09	0.05	0.03	-0.02	0.08	-0.05	0.99
Left amygdala	0.06	0.01	0.10	0.07	0.04	0.10	-0.01	0.99
Left accumbens	-0.05	-0.08	-0.02	-0.01	-0.03	0.01	-0.04	0.57
Right thalamus	0.14	0.00	0.27	0.15	0.06	0.24	-0.02	0.99
Right caudate	-0.21	-0.31	-0.10	-0.13	-0.20	-0.06	-0.08	0.99
Right putamen	-0.10	-0.21	0.02	-0.01	-0.09	0.07	-0.09	0.99
Right pallidum	0.10	0.04	0.16	0.05	0.01	0.09	0.04	0.99
Right hippocampus	-0.08	-0.15	-0.01	-0.01	-0.06	0.04	-0.07	0.99
Right amygdala	0.01	-0.03	0.05	0.04	0.01	0.06	-0.03	0.99
Right accumbens	-0.01	-0.03	0.01	-0.02	-0.03	0.00	0.00	0.99
Left banks superior temporal sulcus	-0.43	-0.59	-0.27	-0.29	-0.41	-0.18	-0.14	0.99
Left caudal anterior cingulate	0.08	-0.07	0.23	0.06	-0.05	0.16	0.02	0.99
Left caudal middle frontal	-0.01	-0.39	0.37	-0.05	-0.31	0.21	0.04	0.99
Left cuneus	-0.26	-0.45	-0.08	-0.16	-0.29	-0.03	-0.10	0.99

Left entorhinal	0.15	-0.05	0.35	-0.07	-0.21	0.07	0.22	0.99
Left fusiform	-0.47	-0.79	-0.15	-0.37	-0.59	-0.15	-0.09	0.99
Left inferior parietal	-1.52	-2.06	-0.98	-1.39	-1.76	-1.01	-0.13	0.99
Left inferior temporal	-0.76	-1.26	-0.25	-0.56	-0.91	-0.22	-0.19	0.99
Left isthmus cingulate	-0.24	-0.38	-0.10	-0.17	-0.27	-0.08	-0.06	0.99
Left lateral occipital	-1.30	-1.83	-0.76	-1.39	-1.76	-1.03	0.10	0.99
Left lateral orbital frontal	-0.08	-0.30	0.15	-0.28	-0.44	-0.13	0.20	0.99
Left lingual	-0.30	-0.61	0.02	-0.20	-0.42	0.02	-0.10	0.99
Left medial orbital frontal	-0.12	-0.29	0.04	-0.41	-0.52	-0.29	0.28	0.47
Left middle temporal	-0.59	-0.96	-0.22	-0.50	-0.76	-0.25	-0.09	0.99
Left parahippocampal	-0.03	-0.17	0.12	0.02	-0.08	0.12	-0.05	0.99
Left paracentral	-0.21	-0.38	-0.03	-0.24	-0.36	-0.12	0.03	0.99
Left pars opercularis	-0.11	-0.37	0.15	-0.14	-0.32	0.03	0.04	0.99
Left pars orbitalis	-0.18	-0.31	-0.06	-0.25	-0.34	-0.17	0.07	0.99
Left pars triangularis	-0.18	-0.35	0.00	-0.21	-0.33	-0.09	0.04	0.99
Left pericalcarine	0.11	-0.05	0.26	0.22	0.11	0.33	-0.11	0.99
Left postcentral	-0.46	-0.90	-0.01	-0.50	-0.81	-0.19	0.04	0.99
Left posterior cingulate	-0.19	-0.34	-0.04	-0.09	-0.19	0.01	-0.10	0.99
Left precentral	0.07	-0.37	0.51	0.05	-0.25	0.35	0.02	0.99
Left precuneus	-0.86	-1.23	-0.48	-1.22	-1.48	-0.96	0.37	0.99
Left rostral anterior cingulate	0.05	-0.10	0.20	0.10	0.00	0.21	-0.05	0.99
Left rostral middle frontal	-0.39	-1.06	0.28	-0.84	-1.30	-0.37	0.45	0.99
Left superior frontal	-0.72	-1.50	0.05	-0.69	-1.23	-0.15	-0.03	0.99

Left superior parietal	-1.89	-2.52	-1.26	-1.55	-1.99	-1.11	-0.34	0.99
Left superior temporal	-1.08	-1.54	-0.62	-0.87	-1.19	-0.55	-0.21	0.99
Left supramarginal	-1.12	-1.72	-0.52	-0.88	-1.29	-0.47	-0.24	0.99
Left frontal pole	-0.26	-0.33	-0.19	-0.22	-0.26	-0.17	-0.04	0.99
Left temporal pole	-0.15	-0.28	-0.01	-0.21	-0.30	-0.11	0.06	0.99
Left transverse temporal	-0.11	-0.18	-0.05	-0.09	-0.14	-0.05	-0.02	0.99
Left insula	-0.34	-0.56	-0.12	-0.40	-0.55	-0.24	0.06	0.99
Right banks superior temporal sulcus	-0.25	-0.38	-0.13	-0.26	-0.34	-0.17	0.00	0.99
Right caudal anterior cingulate	0.13	-0.04	0.30	0.03	-0.09	0.14	0.11	0.99
Right caudal middle frontal	0.06	-0.33	0.44	0.00	-0.26	0.27	0.05	0.99
Right cuneus	-0.33	-0.53	-0.14	-0.27	-0.41	-0.13	-0.06	0.99
Right entorhinal	0.13	-0.06	0.32	0.04	-0.09	0.17	0.09	0.99
Right fusiform	-0.44	-0.78	-0.11	-0.37	-0.60	-0.13	-0.08	0.99
Right inferior parietal	-0.97	-1.66	-0.29	-1.34	-1.81	-0.87	0.37	0.99
Right inferior temporal	-0.51	-1.09	0.07	-0.47	-0.87	-0.07	-0.04	0.99
Right isthmus cingulate	-0.17	-0.31	-0.04	-0.27	-0.36	-0.18	0.10	0.99
Right lateral occipital	-1.80	-2.42	-1.18	-1.43	-1.86	-1.00	-0.37	0.99
Right lateral orbital frontal	-0.47	-0.71	-0.24	-0.49	-0.65	-0.33	0.02	0.99
Right lingual	-0.60	-0.95	-0.26	-0.64	-0.88	-0.41	0.04	0.99
Right medial orbital frontal	-0.52	-0.68	-0.35	-0.41	-0.53	-0.29	-0.11	0.99
Right middle temporal	-0.66	-1.05	-0.26	-0.53	-0.80	-0.25	-0.13	0.99
Right parahippocampal	0.02	-0.08	0.11	0.02	-0.05	0.08	0.00	0.99
Right paracentral	-0.30	-0.48	-0.11	-0.13	-0.26	0.00	-0.17	0.99

Right pars opercularis	-0.07	-0.29	0.15	-0.09	-0.24	0.06	0.02	0.99
Right pars orbitalis	-0.05	-0.18	0.08	-0.25	-0.34	-0.16	0.20	0.54
Right pars triangularis	-0.17	-0.40	0.06	-0.26	-0.42	-0.10	0.09	0.99
Right pericalcarine	0.17	0.00	0.33	0.32	0.21	0.43	-0.16	0.99
Right postcentral	-0.46	-0.89	-0.03	-0.64	-0.93	-0.34	0.18	0.99
Right posterior cingulate	0.00	-0.16	0.15	-0.06	-0.16	0.05	0.05	0.99
Right precentral	0.31	-0.18	0.80	0.43	0.09	0.77	-0.12	0.99
Right precuneus	-1.05	-1.43	-0.67	-1.37	-1.64	-1.11	0.32	0.99
Right rostral anterior cingulate	0.08	-0.05	0.21	0.04	-0.05	0.12	0.04	0.99
Right rostral middle frontal	-0.68	-1.47	0.10	-1.04	-1.59	-0.50	0.36	0.99
Right superior frontal	-0.61	-1.39	0.17	-0.23	-0.77	0.31	-0.38	0.99
Right superior parietal	-1.10	-1.69	-0.52	-1.61	-2.01	-1.20	0.50	0.99
Right superior temporal	-0.85	-1.31	-0.39	-0.82	-1.14	-0.50	-0.03	0.99
Right supramarginal	-0.61	-1.07	-0.14	-0.73	-1.05	-0.40	0.12	0.99
Right frontal pole	-0.27	-0.36	-0.19	-0.27	-0.33	-0.21	0.00	0.99
Right temporal pole	-0.07	-0.20	0.07	-0.25	-0.35	-0.16	0.19	0.57
Right transverse temporal	-0.08	-0.13	-0.03	-0.07	-0.11	-0.04	0.00	0.99
Right insula	-0.04	-0.25	0.17	-0.04	-0.18	0.11	0.00	0.99

Regression coefficients (β) and p-values (false discovery rate corrected) were derived from linear mixed effects models adjusted for sex. Positive β indicate brain volumes increased over time; negative β indicate brain volumes reduced over time. Positive interaction β indicate brain volumes increased more over time in those with neonatal sepsis compared with those without neonatal sepsis; negative interaction β indicate brain volumes increased less over time in those with neonatal sepsis compared with those without neonatal sepsis. N/A= not applicable, CI= confidence interval.

Supplementary Table 2. Descriptive summaries (mean, SD) and differences in mean brain volumes at 13 years of age between very preterm children who did and did not have neonatal sepsis.

Brain volume (cm^3)	Sepsis, *n*=46		No sepsis, *n*=46		Estimates adjusted for sex, GA, BWSDS				Estimates adjusted for sex, GA, BWSDS, WMI, PNCS			
	mean	SD	mean	SD	β	95% CI lower	95% CI upper	*p*	β	95% CI lower	95% CI upper	*p*
Intracranial	1422	144	1446	126	8.30	-32.8	49.4	0.98	18.0	-16.9	52.9	0.94
Total brain tissue	1200	117	1220	120	-3.69	-40.5	33.1	0.98	5.54	-27.0	38.1	0.94
Extra-axial cerebrospinal fluid	202	44.7	205	38.5	15.8	3.85	27.8	0.68	9.63	-3.40	22.7	0.94
Cerebrospinal fluid	222	53.8	226	44.4	18.8	1.43	36.2	0.68	9.66	-7.02	26.3	0.94
Total cortical grey matter	560	54.9	568	55.7	-8.30	-28.6	12.0	0.98	-2.84	-18.2	12.5	0.94
Left cortical grey matter	280	28.4	284	28.0	-3.39	-12.9	6.10	0.98	-0.72	-8.42	6.97	0.94
Right cortical grey matter	280	26.6	284	27.7	-4.97	-16.3	6.35	0.98	-2.16	-10.2	5.84	0.94
Left lateral ventricle	8.83	7.52	8.58	6.08	0.46	-2.08	2.99	0.98	0.11	-2.30	2.52	0.94
Right lateral ventricle	8.20	7.14	8.30	5.59	0.13	-2.28	2.55	0.98	-0.20	-2.57	2.17	0.94
Third ventricle	0.93	0.39	0.91	0.37	0.02	-0.11	0.15	0.98	0.01	-0.12	0.14	0.94
Fourth ventricle	1.93	0.55	1.93	0.62	0.01	-0.18	0.21	0.98	0.02	-0.18	0.22	0.94
Left white matter	190	23.4	196	24.1	-1.13	-8.20	5.94	0.98	0.45	-6.54	7.43	0.94
Right white matter	190	23.3	195	23.9	-1.07	-8.24	6.10	0.98	0.42	-6.53	7.37	0.94
Total white matter	380	46.6	391	47.9	-2.19	-16.4	12.0	0.98	0.87	-13.00	14.74	0.94
Corpus callosum	3.18	0.56	3.32	0.61	0.04	-0.15	0.22	0.98	0.06	-0.14	0.25	0.94
Brainstem	22.1	2.44	22.7	2.48	-0.10	-0.95	0.75	0.98	-0.06	-0.83	0.71	0.94
Left cerebellum	69.8	9.11	70.4	7.38	1.06	-1.71	3.83	0.98	1.51	-1.29	4.31	0.94
Right cerebellum	69.7	9.01	71.0	7.46	0.47	-2.26	3.19	0.98	1.10	-1.53	3.74	0.94
Left thalamus	7.39	0.89	7.48	0.79	0.05	-0.24	0.34	0.98	0.11	-0.18	0.40	0.94
Left caudate	3.54	0.55	3.73	0.50	-0.09	-0.26	0.09	0.98	-0.07	-0.25	0.11	0.94

Left putamen	4.89	0.58	5.01	0.66	-0.07	-0.28	0.14	0.98	-0.07	-0.28	0.14	0.94
Left pallidum	1.93	0.21	2.05	0.23	-0.06	-0.13	0.01	0.87	-0.06	-0.13	0.01	0.94
Left hippocampus	3.20	0.32	3.29	0.33	-0.06	-0.18	0.06	0.98	-0.02	-0.14	0.09	0.94
Left amygdala	1.60	0.14	1.63	0.19	-0.02	-0.07	0.04	0.98	-0.02	-0.07	0.03	0.94
Left accumbens	0.46	0.08	0.47	0.09	-0.01	-0.03	0.02	0.98	-0.01	-0.04	0.02	0.94
Right thalamus	6.96	0.69	7.13	0.81	-0.03	-0.29	0.23	0.98	0.03	-0.22	0.27	0.94
Right caudate	3.60	0.55	3.81	0.50	-0.12	-0.31	0.08	0.98	-0.08	-0.26	0.10	0.94
Right putamen	5.06	0.51	5.22	0.60	-0.12	-0.32	0.08	0.98	-0.08	-0.27	0.11	0.94
Right pallidum	1.81	0.23	1.87	0.23	-0.02	-0.09	0.06	0.98	-0.02	-0.09	0.06	0.94
Right hippocampus	3.27	0.33	3.36	0.30	-0.05	-0.17	0.07	0.98	-0.01	-0.12	0.10	0.94
Right amygdala	1.74	0.15	1.76	0.20	-0.01	-0.07	0.05	0.98	0.00	-0.06	0.06	0.98
Right accumbens	0.58	0.10	0.58	0.10	0.00	-0.03	0.04	0.98	0.01	-0.03	0.04	0.94
Left banks superior temporal sulcus	2.66	0.50	2.74	0.63	0.01	-0.17	0.19	0.98	0.04	-0.14	0.22	0.94
Left caudal anterior cingulate	2.17	0.67	2.24	0.59	-0.01	-0.23	0.22	0.98	0.07	-0.15	0.29	0.94
Left caudal middle frontal	8.01	1.73	7.82	1.19	0.17	-0.44	0.79	0.98	0.28	-0.36	0.92	0.94
Left cuneus	3.60	0.70	3.74	0.61	-0.15	-0.37	0.08	0.98	-0.12	-0.34	0.11	0.94
Left entorhinal	2.36	0.72	2.17	0.64	0.18	-0.08	0.43	0.98	0.24	-0.03	0.50	0.94
Left fusiform	10.2	1.43	10.6	1.45	-0.33	-0.87	0.21	0.98	-0.23	-0.72	0.27	0.94
Left inferior parietal	14.0	2.26	14.1	2.27	0.10	-0.63	0.83	0.98	0.25	-0.47	0.98	0.94
Left inferior temporal	13.9	1.98	14.1	2.19	-0.17	-0.84	0.50	0.98	-0.11	-0.75	0.52	0.94
Left isthmus cingulate	3.14	0.61	3.17	0.58	0.01	-0.19	0.21	0.98	0.04	-0.14	0.23	0.94
Left lateral occipital	14.2	2.33	14.3	2.17	0.00	-0.76	0.77	1.00	0.29	-0.46	1.03	0.94
Left lateral orbital frontal	9.20	1.03	9.18	1.10	0.06	-0.28	0.40	0.98	0.09	-0.25	0.44	0.94

Left lingual	7.18	1.15	7.68	1.24	-0.44	-0.90	0.02	0.79	-0.35	-0.76	0.05	0.94
Left medial orbital frontal	5.86	0.73	5.81	0.78	0.06	-0.22	0.33	0.98	0.09	-0.15	0.34	0.94
Left middle temporal	12.8	1.71	12.9	1.84	0.18	-0.40	0.76	0.98	0.32	-0.30	0.93	0.94
Left parahippocampal	2.30	0.54	2.47	0.49	-0.22	-0.41	-0.03	0.68	-0.18	-0.38	0.02	0.94
Left paracentral	4.26	0.66	4.19	0.63	0.13	-0.11	0.37	0.98	0.13	-0.12	0.38	0.94
Left pars opercularis	5.73	0.88	5.67	1.02	0.14	-0.19	0.47	0.98	0.24	-0.08	0.56	0.94
Left pars orbitalis	2.91	0.39	2.81	0.43	0.13	-0.02	0.28	0.87	0.13	-0.02	0.28	0.94
Left pars triangularis	4.33	0.66	4.38	0.71	0.04	-0.19	0.26	0.98	0.06	-0.18	0.30	0.94
Left pericalcarine	2.54	0.53	2.72	0.48	-0.21	-0.40	-0.02	0.68	-0.24	-0.43	-0.04	0.94
Left postcentral	11.2	1.93	11.1	1.81	0.16	-0.48	0.80	0.98	0.26	-0.38	0.89	0.94
Left posterior cingulate	3.59	0.65	3.77	0.65	-0.13	-0.37	0.12	0.98	-0.11	-0.36	0.14	0.94
Left precentral	15.5	2.05	15.6	1.90	0.11	-0.55	0.77	0.98	0.14	-0.59	0.87	0.94
Left precuneus	12.0	1.72	12.2	1.47	-0.03	-0.53	0.48	0.98	0.15	-0.32	0.62	0.94
Left rostral anterior cingulate	3.22	0.68	3.30	0.63	-0.10	-0.30	0.11	0.98	-0.10	-0.29	0.08	0.94
Left rostral middle frontal	21.3	2.85	21.1	3.11	0.28	-0.61	1.16	0.98	0.39	-0.54	1.32	0.94
Left superior frontal	28.5	3.42	28.4	3.41	0.22	-0.91	1.35	0.98	0.33	-0.86	1.53	0.94
Left superior parietal	14.9	2.05	15.8	2.43	-0.77	-1.44	-0.11	0.68	-0.71	-1.35	-0.07	0.94
Left superior temporal	14.8	2.02	15.1	2.26	0.00	-0.62	0.61	1.00	0.18	-0.45	0.80	0.94
Left supramarginal	13.0	2.38	13.7	2.48	-0.38	-1.04	0.29	0.98	-0.23	-0.93	0.47	0.94
Left frontal pole	1.15	0.18	1.17	0.19	-0.05	-0.12	0.03	0.98	-0.03	-0.11	0.05	0.94
Left temporal pole	2.51	0.35	2.41	0.36	0.11	-0.02	0.24	0.87	0.14	0.00	0.27	0.94
Left transverse temporal	1.36	0.27	1.39	0.29	-0.01	-0.10	0.07	0.98	-0.01	-0.10	0.08	0.94
Left insula	7.80	0.88	7.98	1.01	-0.06	-0.34	0.23	0.98	-0.06	-0.35	0.23	0.94

Right banks superior temporal sulcus	2.26	0.47	2.41	0.45	-0.06	-0.24	0.12	0.98	-0.03	-0.22	0.16	0.94
Right caudal anterior cingulate	2.53	0.76	2.50	0.65	0.05	-0.23	0.32	0.98	0.06	-0.23	0.36	0.94
Right caudal middle frontal	7.57	1.89	7.42	1.16	0.23	-0.42	0.88	0.98	0.36	-0.29	1.00	0.94
Right cuneus	3.99	0.61	4.01	0.70	-0.02	-0.25	0.21	0.98	0.02	-0.18	0.23	0.94
Right entorhinal	2.40	0.70	2.29	0.66	0.08	-0.17	0.33	0.98	0.13	-0.13	0.39	0.94
Right fusiform	9.98	1.34	10.37	1.52	-0.27	-0.72	0.18	0.98	-0.17	-0.61	0.27	0.94
Right inferior parietal	17.4	2.51	17.5	3.06	-0.10	-0.98	0.78	0.98	0.07	-0.81	0.96	0.94
Right inferior temporal	14.3	2.40	14.3	2.28	-0.18	-0.91	0.55	0.98	-0.15	-0.91	0.61	0.94
Right isthmus cingulate	2.74	0.56	2.76	0.48	-0.01	-0.24	0.21	0.98	-0.02	-0.25	0.20	0.94
Right lateral occipital	14.6	2.52	14.5	2.24	0.17	-0.53	0.86	0.98	0.25	-0.46	0.96	0.94
Right lateral orbital frontal	8.61	0.93	8.75	1.07	-0.07	-0.37	0.23	0.98	-0.05	-0.36	0.26	0.94
Right lingual	7.77	1.27	7.98	1.41	-0.08	-0.59	0.44	0.98	-0.03	-0.53	0.48	0.94
Right medial orbital frontal	6.32	0.76	6.52	0.75	-0.15	-0.43	0.13	0.98	-0.08	-0.35	0.18	0.94
Right middle temporal	13.2	1.76	13.7	1.74	-0.33	-0.88	0.21	0.98	-0.26	-0.81	0.29	0.94
Right parahippocampal	1.99	0.33	2.14	0.35	-0.12	-0.25	0.00	0.76	-0.09	-0.22	0.04	0.94
Right paracentral	4.62	0.63	4.79	0.70	-0.12	-0.34	0.11	0.98	-0.15	-0.36	0.07	0.94
Right pars opercularis	4.70	0.72	4.74	0.80	0.05	-0.22	0.32	0.98	0.12	-0.16	0.40	0.94
Right pars orbitalis	3.49	0.40	3.36	0.45	0.13	-0.03	0.29	0.87	0.13	-0.04	0.30	0.94
Right pars triangularis	4.97	0.67	5.01	0.81	0.02	-0.27	0.30	0.98	0.06	-0.21	0.32	0.94
Right pericalcarine	2.92	0.61	3.15	0.66	-0.24	-0.48	0.00	0.76	-0.22	-0.46	0.02	0.94
Right postcentral	10.3	1.83	10.7	1.70	-0.03	-0.66	0.59	0.98	0.13	-0.45	0.70	0.94
Right posterior cingulate	3.69	0.76	3.76	0.63	0.02	-0.22	0.27	0.98	0.07	-0.19	0.33	0.94
Right precentral	15.3	2.03	15.6	1.76	-0.10	-0.77	0.56	0.98	-0.16	-0.86	0.54	0.94

Right precuneus	12.5	1.54	12.6	1.66	0.12	-0.42	0.65	0.98	0.28	-0.17	0.74	0.94
Right rostral anterior cingulate	2.30	0.40	2.34	0.49	-0.06	-0.23	0.10	0.98	-0.07	-0.24	0.11	0.94
Right rostral middle frontal	21.1	2.62	21.2	3.07	-0.02	-0.98	0.94	0.99	0.24	-0.72	1.20	0.94
Right superior frontal	27.4	3.46	27.8	3.55	-0.08	-1.07	0.92	0.98	0.05	-0.98	1.08	0.94
Right superior parietal	15.0	1.89	15.2	2.31	-0.29	-1.11	0.53	0.98	-0.27	-1.16	0.61	0.94
Right superior temporal	13.6	1.98	13.9	1.96	-0.05	-0.73	0.63	0.98	0.12	-0.58	0.82	0.94
Right supramarginal	11.8	1.95	12.0	1.90	0.02	-0.69	0.73	0.98	0.11	-0.63	0.85	0.94
Right frontal pole	1.30	0.20	1.30	0.20	-0.02	-0.09	0.05	0.98	-0.02	-0.10	0.05	0.94
Right temporal pole	2.58	0.42	2.51	0.38	0.12	-0.03	0.27	0.87	0.14	-0.02	0.29	0.94
Right transverse temporal	1.05	0.19	1.04	0.19	0.00	-0.07	0.07	0.98	0.01	-0.07	0.08	0.94
Right insula	7.80	0.93	8.01	0.95	-0.08	-0.41	0.25	0.98	-0.09	-0.43	0.25	0.94

Regression coefficients (β) and their corresponding *p*-values (false discovery rate corrected) represent mean differences in brain volumes between those who did and did not have neonatal sepsis, and were derived from linear mixed effects models. Positive β indicate higher volumes in those who did have neonatal sepsis; negative β indicate lower volumes in those who did have neonatal sepsis. BWSDS= birthweight standard deviation score, CI= confidence interval, GA= gestational age, N/A= not applicable, PNCS= postnatal corticosteroid exposure, SD = standard deviation, WMI= white matter injury.

Supplementary Table 3. Descriptive summaries (mean, SD) and differences in mean (β) neurodevelopmental outcomes at 13 years of age between very preterm children who did and did not have neonatal sepsis, adjusted for covariates.

	Sepsis, *n*=63		No sepsis, *n*=114		Estimates adjusted for sex, GA, BWSDS				Estimates adjusted for sex, GA, BWSDS, WMI, PNCS			
Outcome	mean	SD	mean	SD	β	95% CI lower	95% CI upper	*p*	β	95% CI lower	95% CI upper	*p*
IQ[a]	99.0	17.6	101	18.7	-0.62	-5.19	3.94	0.86	0.20	-4.25	4.66	0.94
Attention[a]	7.98	3.87	8.14	3.51	0.11	-1.08	1.30	0.86	0.15	-1.02	1.32	0.94
Working memory	89.1	13.9	92.9	14.0	-3.96	-9.09	1.18	0.86	-2.83	-7.84	2.18	0.94
Memory & learning	46.8	12.2	48.0	11.5	-2.12	-6.13	1.88	0.86	-1.38	-5.29	2.52	0.94
Language[b]	98.6	16.7	99.6	18.2	-0.87	-4.42	2.67	0.86	-0.13	-3.41	3.15	0.94

Executive function[c]	10.0	2.44	10.1	2.09	-1.58	-6.56	3.39	0.86	-1.22	-5.94	3.50	0.94
Reading[a]	101	17.2	103	17.0	-0.08	-0.76	0.61	0.86	0.10	-0.55	0.76	0.94
Spelling[d]	101	18.8	105	19.3	-3.30	-8.44	1.83	0.86	-2.10	-7.13	2.94	0.94
Mathematics[e]	89.9	14.6	93.0	16.7	-2.92	-8.89	3.05	0.85	-2.18	-8.27	3.91	0.94
Behavior[f]	11.1	6.55	10.5	5.65	-3.31	-7.26	0.64	0.86	-2.01	-5.55	1.54	0.94
Motor skills[g]	9.79	7.97	8.89	5.83	0.43	-1.53	2.38	0.86	-0.09	-1.97	1.78	0.94

Regression coefficients (β) and their corresponding *p*-values (false discovery rate corrected) represent mean differences in outcome scores between the children who did and did not have neonatal sepsis, and were derived from linear regression models adjusting for variables as listed. Positive β indicate higher mean outcome scores in those who did not have neonatal sepsis; negative β indicate lower mean outcome scores in those who did have neonatal sepsis. Note: For all scores higher values indicate more favorable outcomes except behavior, where higher values indicate a less favorable outcome. [a]*n*=62 in Sepsis group, [b]*n*=55 in Sepsis group, *n*=106 in No sepsis group, [c]*n*=61 in Sepsis group, *n*=112 in No sepsis group, [d]*n*=60 in Sepsis group, [e]*n*=61 in Sepsis group, [f]*n*=43 in Sepsis group, *n*=84 in No sepsis group, [g]*n*=56 in Sepsis group, *n*=107 in No sepsis group. GA= gestational age, BWSDS= birthweight standard deviation score, SD = standard deviation, WMI= white matter injury, PNCS= postnatal corticosteroid exposure.
